# Lactated Ringer Versus Normal Saline for Fluid Resuscitation in Acute Pancreatitis: A Systematic Review

**DOI:** 10.7759/cureus.117857

**Published:** 2026-10-13

**Authors:** Aditya Lather, Yashika Singh Chauhan, Sonia Phogat, Pardeep Kumar, Mete Emre Ozer, Aleksei Markin, Jai Behgal, Tanvi Lather, Lubna Mohammed

**Affiliations:** 1 Internal Medicine, All India Institute of Medical Sciences, Patna, IND; 2 Obstetrics and Gynaecology, Kerala University of Health Sciences, Palakkad, IND; 3 Internal Medicine, Dr. Baba Saheb Ambedkar Medical College and Hospital, Delhi, IND; 4 General Medicine, Pandit Bhagwat Dayal Sharma Postgraduate Institute of Medical Sciences, Rohtak, IND; 5 Health, Ministry of Health, Kirikkale, TUR; 6 Internal Medicine, Sechenov University, Moscow, RUS; 7 Internal Medicine, University of Louisville Hospital, Louisville, USA; 8 Principles and Practice of Clinical Research, Harvard School of Public Health, Boston, USA

**Keywords:** acute pancreatitis (ap), intravenous fluid therapy, lactated ringer, normal saline (ns), sirs persistence

## Abstract

Acute pancreatitis (AP) requires early fluid resuscitation, and the optimal choice of fluid remains uncertain. This review compared lactated Ringer (LR) with normal saline (NS) in adult AP, with a primary focus on systemic inflammatory response syndrome (SIRS) at 24 hours*. *Following Preferred Reporting Items for Systematic Reviews and Meta-Analyses (PRISMA) 2020, this review explored PubMed, ScienceDirect, ClinicalTrials.gov, Europe PMC, EBSCO Open Dissertations, and the Cochrane Library for studies published from January 1, 2015 to October 31, 2025. We screened titles and abstracts of 1,324 records and 14 full-text reports. Out of the 14 reports, six met the inclusion criteria of our review. This included three small randomized controlled trials (RCTs) and three meta-analyses (MAs). Throughout the review, LR was consistently associated with lower rates of intensive care unit (ICU) admissions. In addition, short-term inflammatory markers - such as SIRS and C-reactive protein (CRP) - tended to demonstrate earlier improvement with LR, while findings related to mortality and length of hospital (LOH) stay were less consistent and often did not reach statistical significance. Early fluid resuscitation is a central part of AP care, yet the optimal crystalloid has remained uncertain. By reviewing recent RCTs and MAs, the review supports that LR is physiologically and clinically better than NS. This review also highlights areas where current evidence remains limited and heterogeneous. Suggestions for future research include conducting a larger, multicenter randomized trial with standardized resuscitation protocols to ensure adequate statistical power. A more extensive trial is needed to establish a definitive conclusion, particularly for outcomes that have mixed or underpowered results in the available literature.

## Introduction and background

Acute pancreatitis (AP) is defined as sudden inflammation of the pancreas. It causes over three lakh hospitalizations annually in the United States alone [1]. Patients with AP often require hospital stays longer than two months. The clinical progression of AP is highly unpredictable, spanning from a mild, self-resolving condition to a critical illness characterized by multi-organ dysfunction and significant mortality rates. The in-hospital mortality rate for AP is 2.8% [2], with mortality for severe AP going as high as 30% [3].

In AP, precursor enzymes of the pancreas are activated prematurely. This leads to autodigestion of the pancreas and the release of inflammatory mediators. These inflammatory mediators, consequently, cause vascular changes, such as vasoconstriction and increased vascular permeability [4,5].

Hence, early fluid resuscitation is the cornerstone of AP management, with guidelines such as those from the American College of Gastroenterology (ACG) recommending Lactated Ringer (LR) over normal saline (NS) [1]. Although colloids were historically used, crystalloids are now the standard fluid choice for resuscitation [6]. However, recommendations regarding specific crystalloids vary across societies: the American Gastroenterological Association (AGA) suggests goal-directed fluid therapy without expressing a preference between NS and LR [7], whereas the Japanese Clinical Practice Guidelines [8], along with the British Society of Gastroenterology (BSG), European Society of Gastrointestinal Endoscopy (ESGE), and International Association of Pancreatology/American Pancreatic Association (IAP/APA) guidelines [9-11], explicitly favor LR or balanced crystalloids over NS. Nevertheless, most guideline preferences toward LR remain conditional, reflecting variations in the strength of underlying evidence across panels.

Although early randomized trials demonstrated that LR reduces systemic inflammation, measured via lower C-reactive protein (CRP) levels, compared to NS [12,13], its impact on clinical outcomes remained less clear. Subsequent reviews have suggested potential secondary benefits in parameters such as length of hospital (LOH) stay and intensive care unit (ICU) admission, but failed to show a definitive reduction in mortality or systemic inflammatory response syndrome (SIRS) resolution [14,15]. Furthermore, the overall certainty of this comparative evidence is limited by small trial sample sizes, clinical heterogeneity, and reliance on observational data.

Therefore, this systematic review aims to evaluate the evidence from the last decade comparing LR and NS in AP, specifically focusing on SIRS as the primary outcome while assessing secondary clinical parameters and the overall certainty of evidence.

## Review

Methods

This systematic review followed the Preferred Reporting Items for Systematic Reviews and Meta-Analyses (PRISMA) 2020 guidelines.

The research question, framed within the relevant PICO (Population, Intervention, Comparison, Outcome) framework, was as follows: The population comprised adults aged 18 years or older with AP. AP is diagnosed when two of the three revised Atlanta classification criteria are met [16]. Intervention included LR, and the control was NS. The primary outcome was prevalence of SIRS at 24 hours. SIRS is defined by the presence of any two of the following criteria: body temperature >100.4°F (38°C) or <96.8°F (36°C), heart rate >90 bpm, respiratory rate >20 breaths/minute or PaCO_2_ <32 mm Hg (4.3 kPa), leukocyte count >12,000/μL, <4,000/μL, or >10% immature forms (bands). Secondary outcomes were the LOH stay, mortality rates, occurrence of moderately severe to severe pancreatitis, and local complications (e.g., pancreatic necrosis, abscess, pseudocyst formation).

Search strategy and selection criteria

A systematic search was independently conducted by two reviewers across PubMed, ScienceDirect, ClinicalTrials.gov, Europe PMC, EBSCO Open Dissertations, and the Cochrane Library to retrieve all original study data (Table 1). Key search terms used included “Acute Pancreatitis”, “Pancreatitis”, “Lactated Ringer”, “Fluid Therapy”, “Normal Saline" and their word variations. We used Boolean operators (AND, OR) to narrow the results. We conducted a comprehensive search in PubMed, ScienceDirect, ClinicalTrials.gov, Europe PMC, EBSCO Open Dissertations, and the Cochrane Library for studies published between January 1, 2015, and October 31, 2025. This initial search identified 2,834 articles.

**Table 1:** Search strategy used for this review

Search strategy	Database used	Number of papers identified
Acute pancreatitis OR pancreatitis ("Pancreatitis/complications"(Majr) OR "Pancreatitis/drug therapy"(Majr) OR "Pancreatitis/metabolism"(Majr) OR "Pancreatitis/mortality"(Majr) OR "Pancreatitis/therapy"(Majr)) AND normal saline OR saline solution OR ("Saline Solution/administration and dosage"(Majr) OR "Saline Solution/therapeutic use"(Majr)) AND Ringer's Lactate OR Lactated ringer OR Hartmann's solution OR ("Ringer's Lactate/administration and dosage"(Majr) OR "Ringer's Lactate/therapeutic use"(Majr) ) AND fluid therapy OR fluid resuscitation	PubMed	817 (filters applied: comparative study, meta-analysis, multicenter study, network meta-analysis, observational study, randomized controlled trial, review, systematic review, English, humans, adult: 19+ years, exclude preprints, from 2015/1-2025/10/31)
((((((acute pancreatitis(Title/Abstract)) OR (pancreatitis(Title/Abstract))) AND (normal saline(Title/Abstract))) OR (saline solution(Title/Abstract))) AND (Ringer's Lactate(Title/Abstract))) OR (Lactated ringer(Title/Abstract))) OR (Hartmann's solution(Title/Abstract))	PubMed (advanced)	119 (filters applied: comparative study, meta-analysis, multicenter study, network meta-analysis, observational study, randomized controlled trial, review, systematic review, English, humans, adult: 19+ years, exclude preprints, from 2015/1-2025/10/31)
((("acute pancreatitis" OR pancreatitis) AND ("normal saline" OR "saline solution") AND ("Lactated ringer" OR "Ringer lactate" OR "Ringer's lactate" OR "Hartmann* solution")) AND FIRST_PDATE:(2015-01-01 to 2025-10-31))	Europe PMC	201
Condition/disease: Acute Pancreatitis OR Pancreatitis Intervention/treatment: Lactated Ringer AND Normal Saline	ClinicalTrials.gov	Eight (completed studies; adults: 18-64; other adults: 65+)
Acute pancreatitis AND Lactated Ringer Solution AND normal saline solution	Cochrane Central Register of Controlled Trials	Six trials (with Cochrane Library publication date from Jan 2015 to Oct 2025 (word variations have been searched))
acute pancreatitis AND ("normal saline" OR "normal saline solution") AND ("lactated ringer" OR "ringer lactate" OR "ringer's lactate" OR "Hartmann solution")	ScienceDirect	298 (English; 2015-2025)
Acute pancreatitis AND Lactated Ringer AND Normal Saline	EBSCO Open Dissertations	Nine

Eligibility criteria

Inclusion Criteria

Papers written and published in the English language; participants were adult males and non-pregnant females aged 18 years or older with AP; studies directly and primarily comparing only LR and NS in the treatment of AP, regardless of the volume administered or the duration of therapy; comparative studies, meta-analyses and systematic reviews, observational studies, and randomized controlled trials (RCTs) were considered; studies published between January 1, 2015, and October 31, 2025.

Exclusion Criteria

Conference abstracts, poster presentations, journal abstracts whose full text could not be retrieved, gray literature, preprints; not written in English; studies involving animals and studies not matched with the PICO elements; critically ill patients with AP or ICU-admitted patients; studies only among patients with post-ERCP pancreatitis or diseases other than AP.

Study selection and data extraction

Titles and abstracts were screened by two independent reviewers using Rayyan software (Qatar Computing Research Institute (QCRI); Doha, Qatar) [17]. Full-text articles of potentially relevant studies were retrieved and assessed against eligibility criteria. Disagreements during screening were resolved through mutual discussions and agreement. Data were extracted using a standardized form. As this is a systematic review of published literature, ethical approval was not required.

A meta-analysis was not performed because the included studies were methodologically heterogeneous, had diverse patient populations, and used different outcome measures. In addition, substantial overlap among primary studies in the included MAs and the modest overall sample size limited the appropriateness of quantitative pooling.

SIRS is an early predictor of severity and the development of organ failure. The prevalence of SIRS measures the inflammatory response you would expect fluid choice to affect, and 24 hours is early enough to capture the anti-inflammatory effect of the fluid. ICU-admitted or critically ill patients were excluded to keep the review focused on the general adult AP population and to avoid confounding from severity and ICU-level interventions.

Quality assessment

For quality assessment, the AMSTAR-2 checklist [18] (Table 2) was used for MAs, and the Cochrane RoB-2 tool [19] (Table 3) was used for RCTs. The MAs were conducted with standard systematic-review methods, i.e., PRISMA adherence, but only two out of three studies had PROSPERO registration, and they were built on a very small pool of trials; one review included only four RCTs, another six studies with 431 patients, and one concluded that “the evidence is mostly of low quality.” For MAs, many items from the AMSTAR-2 checklist were rated “no”; hence, the overall quality of evidence was low to moderate. The RCTs also showed mixed risk of bias: one was double-blinded, which lowered the likelihood of performance and detection bias, while the others were small, single-center or open-label studies, and one was registered only after enrolment had already begun, which raises concerns about selection and reporting bias.

**Table 2:** AMSTAR-2 checklist [18] for meta-analyses The AMSTAR-2 checklist comprises 16 distinct domains. The AMSTAR-2 tool evaluates overall quality by assessing performance in each of these domains. All domains were answered as “yes” or “no” in AMSTAR-2. “Partial yes” was allowed in some domains to identify partial adherence to standard protocols. PICO: population, intervention, control, and outcomes

Checklist questions	Wang et al. [20]	Zhou et al. [21]	Mosquera et al. [22]
Did the review ask a clear PICO question and define inclusion criteria?	Yes	Yes	Yes
Was a protocol registered before the review began, with justified deviations?	No	No	Partial yes
Did the authors justify the study designs they included?	No	No	No
Did they use a comprehensive literature search?	Yes	Yes	Yes
Was study selection done in duplicate?	Yes	Yes	Yes
Was data extraction done in duplicate?	Yes	Yes	Yes
Did they list excluded full-text studies and justify exclusions?	No	No	No
Did they describe the included studies in enough detail?	Yes	Yes	Yes
Did they assess the risk of bias appropriately in the included studies?	Yes	Yes	Yes
Did they report funding sources for the included studies?	No	No	No
If a meta-analysis was done, was the statistical synthesis appropriate?	Yes	Yes	Yes
If a meta-analysis was done, did they assess the impact of risk of bias on the synthesis?	No	No	Yes
Did they account for the risk of bias when interpreting the results?	Yes	Yes	Yes
Did they explain any heterogeneity?	Yes	Yes	Yes
If they synthesized quantitatively, did they assess publication bias?	Yes	Yes	No
Did they report conflicts of interest and funding for the review itself?	Yes	Yes	Yes

**Table 3:** Cochrane RoB-2 tool [19] for RCTs Cochrane RoB-2 tool evaluates the risk of bias in RCTs across five key domains. Each domain is rated as either "Low," "Some concerns," or "High." The domains are as follows: (1) bias arising from the randomization process, (2) bias due to deviations from intended interventions, (3) bias due to missing outcome data, (4) bias in measurement of the outcome, and (5) bias in selection of the reported result. The highest level of risk is categorized as "High," while the lowest is classified as "Low." RCT: randomized controlled trial

RCTs	Domain-1	Domain-2	Domain-3	Domain-4	Domain-5	Overall risk
Lee et al. [23]	Low	Low	Some concerns	Low	Low	Low
Choosakul et al. [24]	Low	Low	Low	Low	Low	Low
Karki et al. [25]	Some concerns	Low	Low	Low	Some concerns	Some concerns

Results

Search Results

The initial search identified 2,834 records. After applying a filter for types of studies (comparative studies, MAs, multicenter studies, network MAs, observational studies, RCTs, reviews, systematic reviews) on PubMed, 1,458 studies were transferred to Rayyan [17].

After removing 134 duplicates, 1,324 records were screened for titles/abstracts, and 31 full-text articles were sought for retrieval. The full text for 17/31 studies was not available.

Eight out of 31 studies were excluded from the pool. The reasons for excluding eight studies were not including SIRS as one of the outcomes (3/8), insufficient databases used for their studies (3/8), different population age (1/8), and duplicates (1/8).

Six studies met the inclusion criteria and were included in the final analysis as illustrated in the PRISMA 2020 flow diagram [26] (Figure 1).

**Figure 1:**
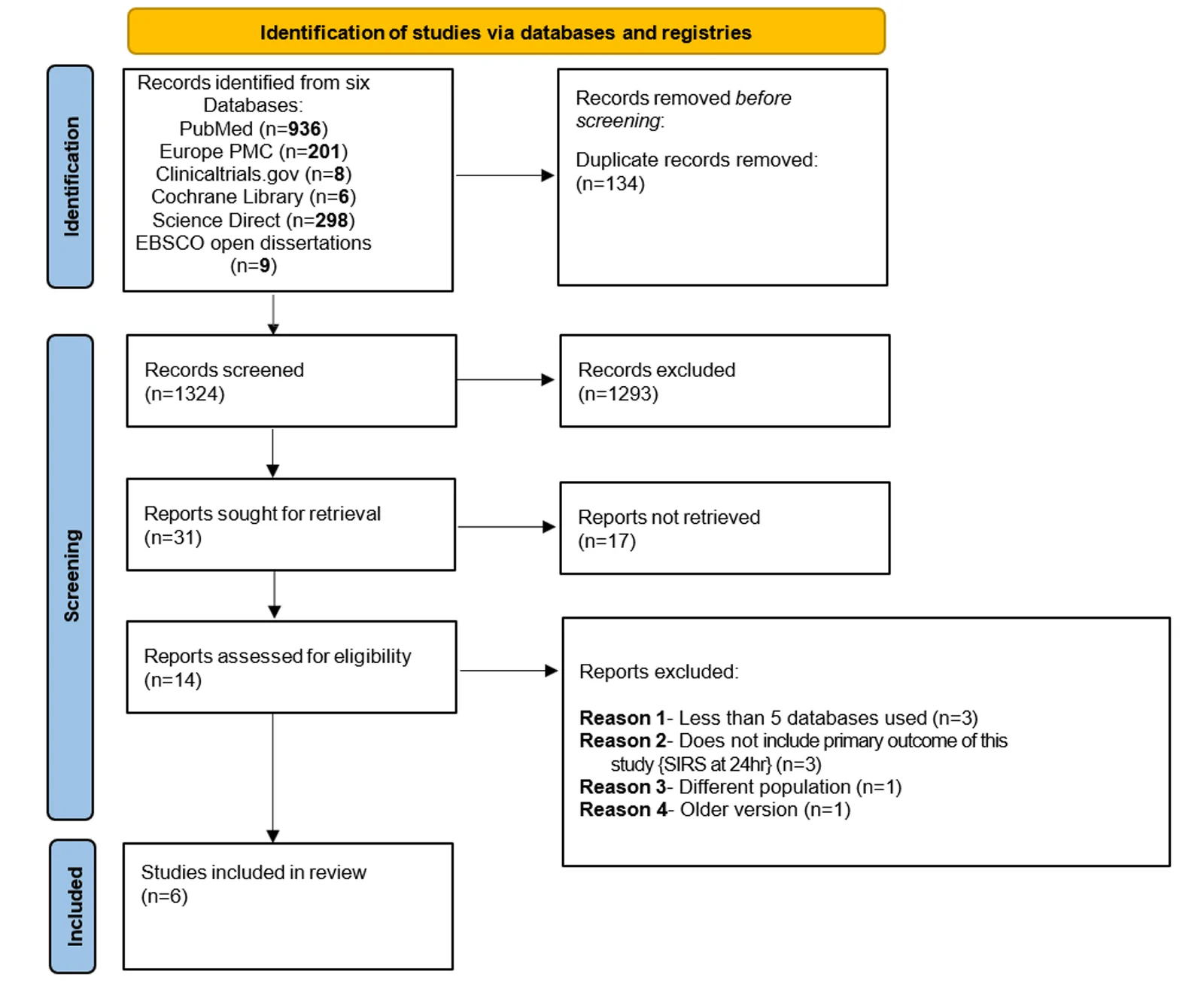
PRISMA 2020 flow diagram PRISMA: Preferred Reporting Items for Systematic Reviews and Meta-Analyses

Study Characteristics

The six included studies all evaluated LR versus NS for the management of AP, but they varied in design and scale, as shown in Table 4. The three included randomized trials represented a cohort of 219 from the USA, Thailand, and Nepal. Sample size ranged from 47 to 121.

**Table 4:** Characteristics of included studies LR: lactated Ringer; NS: normal saline; SIRS: systemic inflammatory response syndrome; CRP: C-reactive protein; ICU: intensive care unit; RCT: randomized controlled trial

	Secondary meta-analysis and systematic review (Tier-2)			Primary randomized controlled trials (Tier-1)		
Author	Wang et al. [20]	Zhou et al. [21]	Mosquera et al. [22]	Lee et al. [23]	Choosakul et al. [24]	Karki et al. [25]
Year	2023	2021	2025	2021	2018	2022
Country	Multiple	Multiple	Multiple	US	Thailand	Nepal
Sample size	431	248	299	121	47	51
Study design	Meta-analysis and systematic review	Meta-analysis and systematic review	Meta-analysis and systematic review	RCT	RCT	RCT
Intervention details	Six RCTs comparing LR with NS fluid resuscitation in acute pancreatitis	Pooled RCT evidence comparing LR fluid resuscitation against NS in acute pancreatitis	Randomized clinical trials of LR versus NS fluid therapy were pooled across databases from 2000 to 2024	1:1 randomization to LR vs NS; both groups received a 10 mL/kg bolus followed by continuous infusion at 3 mL/kg per hour, with blinded assessments at 12, 24, 48, and 72 hours.	Randomized to NS or LR through a goal-directed fluid resuscitation protocol.	Randomized to LR or NS and received the assigned fluid resuscitation protocol for three days.
Outcomes measured	SIRS at 24 hours, length of hospitalization, moderately severe/severe AP, ICU admission, local complications, and pancreatic necrosis.	Primary: development of moderately severe/severe pancreatitis. Additional outcomes: local complications, ICU admission, length of stay, mortality, SIRS, organ failure, and volume overload.	ICU admission, progression of pancreatitis, mortality, hospital stay, SIRS at 24/48/72 hours, and CRP at 48/72 hours.	Primary: change in SIRS prevalence at 24 hours. Secondary: SIRS at 48 and 72 hours, moderately severe/severe pancreatitis, ICU admission, length of hospitalization, and adverse events.	SIRS at 24 and 48 hours, mortality, local complications, inflammatory markers, and length of hospital stay.	SIRS scores and CRP level assessed at baseline and after treatment.

Because the three included MAs contained overlapping subsets of the primary RCTs as detailed in Table 5, findings from secondary MAs were analyzed to compare pooled effect estimates rather than counted as additional independent patient cohorts. Besides the overlap of primary RCTs in secondary MAs, there was also overlap of other RCTs amongst secondary MAs. Thus, the finalized studies were structured into two distinct tiers as follows: Tier 1 (primary RCTs); three primary trials (n = 219, unique patients) evaluated direct, unpooled individual patient outcomes. Tier 2 (secondary MAs); three systematic reviews/MAs, pooling four to six RCTs (n = 431, different patients).

**Table 5:** Overlap matrix of included primary RCTs across secondary meta-analyses RCT: randomized controlled trial

Primary RCT	Included in Wang et al. [20]	Included in Zhou et al. [21]	Included in Mosquera et al. [22]
Lee et al. [23]	Yes	Yes	Yes
Choosakul et al. [24]	Yes	Yes	Yes
Karki et al. [25]	Yes	No	Yes

All primary RCTs were small, single-center adult AP studies comparing LR with NS, but they differed in blinding and protocol intensity. The Thai study was a prospective randomized trial in Bangkok with 47 patients, the US study was double-blinded in Los Angeles with 121 randomized patients, and the Nepalese study was open-label with 51 enrolled patients.

The three secondary MAs pooled four to six RCTs and 248 to 431 patients. Each used PRISMA-based searches and restricted inclusion to randomized trials. All focused on adult AP and compared LR with NS, with overlapping outcomes such as SIRS, progression to moderately severe/severe disease, ICU admission, length of stay, local complications, and mortality.

Given the heterogeneity in study designs, the diverse populations examined, and the variety of outcomes assessed, an MA was not performed.

Synthesis of Outcome

SIRS prevalence at 24 hours: All six included studies reported the prevalence of SIRS after 24 hours of fluid resuscitation. Primary RCTs (Tier 1): In the prospective RCT by Choosakul et al., LR significantly increased the likelihood of SIRS resolution compared to NS (RR = 1.96, 95% confidence interval (CI): 1.22-3.16, P = 0.02), with significantly fewer patients retaining a SIRS score 1 or more in the LR group (17.4%, 4/23) compared to the NS group (45.8%, 11/24, P = 0.025). In Karki et al., none of the 26 patients in the LR group presented with SIRS at 24 hours compared to 2 of 25 patients in the NS group. Conversely, Lee et al. observed no statistically significant difference between groups (adjusted RR = 1.1, 95% CI: 0.7-1.6), which was attributed to a lower baseline SIRS prevalence (26%) in their trial cohort. Secondary MAs (Tier 2): Wang et al. initially observed substantial heterogeneity across trials (RR = 0.63, 95%CI: 0.28-1.43, P = 0.27; I² = 63%); however, upon removing the source of heterogeneity in a sensitivity analysis, LR demonstrated a statistically significant reduction in 24-hour SIRS (RR = 0.43, 95%CI: 0.22-0.82, P = 0.01, I² = 36%). In contrast, pooled estimates in Zhou et al. (odds ratio (OR) = 0.6, 95%CI: 0.22-1.62, I² = 47.3) and Mosquera et al. (RR = 0.59, 95%CI: 0.23-1.49, P = 0.19) showed non-significant trends favoring LR.

SIRS prevalence at 48 hours: Five studies evaluated SIRS persistence at 48 hours, with none demonstrating statistically significant differences between LR and NS, as shown in Table 6.

**Table 6:** Outcomes of studies SIRS: systemic inflammatory response syndrome; LOH: length of hospital; CRP: C-reactive protein; CI: confidence interval; RR: relative risk; OR: odds ratio; NS: normal saline; LR: lactated Ringer; MD: mean difference; SMD: standardized mean difference; IQR: interquartile range; NR: not reported

	Secondary meta-analysis and systematic review (Tier-2)			Primary randomized controlled trials (Tier-1)		
Author	Wang et al. [20]	Zhou et al. [21]	Mosquerra et al. [22]	Lee at al. [23]	Choosakul et al. [24]	Karki et al. [25]
SIRS at 24 hours	RR = 0.63, 95%CI: 0.28-1.43, P = 0.27; I² = 63% After eliminating the source of heterogeneity RR = 0.43, 95%CI: 0.22-0.82, P=0.01 I²=36%	OR = 0.6, 95%CI: 0.22-1.62, I² = 47.3	RR = 0.59, 95% CI: 0.23-1.49, P = 0.19	Adjusted RR = 1.1, CI95%: 0.7-1.6	RR = 1.96, CI 95%: 1.22-3.16, P = 0.02	LR: 0 patient, NS: two patients SIRS score 1 or more LR: four patients, NS: 11 patients. P = 0.025
LOH stay	MD = -0.74, 95%CI: -1.20 to -0.28, P = 0.001	(SMD): -0.18, 95%CI: -0.44-0.07, I² = 0.0	SMD: -0.89; 95% CI: -2.26 to 0.48; P = 0.23)	{Median days (IQR)) LR: {3.5 (2.0-5.9)} NS: {4.6 (2.95-7.35)) (P = 0.049)	{Median days (IQR) LR: (6(4-9)) NS: {5.5(4-9)) (P = 0.915)	(Mean (SD)) LR: {5.15 (0.09)) NS: (6.20 (2.5)) (P = 0.06)
Progression to moderate-severe/severe	RR = 0.64, 95%CI: 0.45-0.89, P = 0.008	OR = 0.49, 95%CI: 0.25-0.97, I² = 0.0	RR = 0.63, 95% CI: 0.40-0.98, P = 0.04	Adjusted RR = 0.5, 95%CI: 0.2-1.1	NR	RL: 23.07% of patients (6/26) NS: 42.30% of patients (11/25) (P = 0.11)
ICU admission rate	RR = 0.39, 95%CI: 0.18-0.85, P = 0.02	OR = 0.33, 95% CI: 0.13-0.81, I² = 0.0	RR = 0.39; 95% CI: 0.18-0.85, P = 0.02	RR = 0.4, 95% CI: 0.2-0.9	NR	NR
Local complications	RR = 0.63, 95%CI: 0.42-0.94, P = 0.02	OR = 0.42, 95%CI: 0.2-0.88, I²=0.0	NR	Adjusted RR = 0.3, 95% CI: 0.1-1.5	LR: 6 (26.08%) NS: 9 (37.5%) P = 0.74	LR: 6 (23.1%) NS: 7 (28%) P = 0.68
SIRS at 48 hours	RR = 0.87, 95%CI: 0.55-1.35, P = 0.53	OR = 0.7, 95%CI: 0.30-1.63, I² = 22.7	RR = 0.83, 95% CI: 0.52-1.34, P = 0.45	Adjusted RR = 1.0, 95%CI: 0.6-1.5	RR = 0.98, 95%CI: 0.35-2.72, P = 1	NR
SIRS at 72 hours	RR = 0.71, 95%CI: 0.41-1.22, P = 0.21	NR	RR = 0.64, 95% CI: 0.35-1.16, P = 0.14	Adjusted RR = 1.0, 95%CI; 0.5-1.8	NR	0 in LR. 0 in NS
Mortality rate	RR = 0.34, 95%CI: 0.06-2.12, P = 0.25	OR = 0.34, 95% CI: 0.03-3.42, I² = 0.0	NR	No mortality	One in NS died	One in NS died, P = 0.9
Change in CRP levels	NR	NR	CRP at 48 hours: SMD = -3.91, 95% CI: -4.66 to −3.17, P < 0.00001 CRP at 72 hours: SMD = -4.92, 95% CI: −17.67 to 7.83, P = 0.13; I² = 81%	NR	NR	CRP at 72 hours The median value of CRP-LR: 14.2. NS: 22.2. P < 0.001

SIRS prevalence at 72 hours: Four studies presented data at the 72-hour mark. Primary RCTs (Tier 1): In Karki et al., 0 patients in both the LR and NS groups exhibited SIRS at 72 hours. In Lee et al., odds of 72-hour SIRS were identical between both resuscitation arms (adjusted RR = 1.0, 95%CI: 0.5-1.8). Secondary MAs (Tier 2): Neither Wang et al. (RR = 0.71, 95%CI: 0.41-1.22, P = 0.21) nor Mosquera et al. (RR = 0.64, 95%CI: 0.35-1.16, P = 0.14) reached statistical significance.

CRP dynamics: Primary RCTs (Tier 1): Karki et al. reported a statistically significant reduction in median CRP levels at 72 hours in the LR group compared to NS (the median value of CRP-LR: 14.2, NS: 22.2, P < 0.001). Secondary MAs (Tier 2): Mosquera et al. demonstrated a statistically significant pooled reduction in CRP levels at 48 hours for LR (SMD = -3.91, 95%CI: -4.66 to -3.17, P < 0.00001). However, this effect lost statistical significance by 72 hours (SMD = -4.92, 95%CI: -17.67 to 7.83, P = 0.13), driven by high inter-study heterogeneity (I² = 81%).

Mortality Rates

Mortality outcomes were generally low across the evidence.

Primary RCTs (Tier 1): Lee et al. reported zero deaths in both arms. Choosakul et al. recorded one death in the NS group (4.17%) and none in the LR group. Karki et al. recorded one death in the NS group (4.0%) and none in the LR group (P = 0.90).

Secondary MAs (Tier 2): Meta-analytic pooling confirmed no statistically significant mortality reduction, as reported by Wang et al. (RR = 0.34, 95%CI: 0.06-2.12, P = 0.25) and Zhou et al. (OR = 0.34, 95%CI: 0.03-3.42, I² = 0.0). In Mosquera et al., the maximum number of deaths in any single included trial was one (in the NS arm).

Length of Hospital Stay

All included studies evaluated hospital duration, showing a general direction of effect favoring LR, though statistical significance varied.

Primary RCTs (Tier 1): Lee et al. demonstrated a statistically significant reduction in median hospital stay for LR compared to NS (3.5 days (IQR: 2.0-5.9)) vs. (4.6 days (IQR: 2.95-7.35))(P = 0.049). Karki et al. noted a non-significant trend toward shorter mean hospital stay with LR (5.15 ± 0.09 days vs. 6.20 ± 2.5 days, P = 0.06). Choosakul et al. found no significant difference in median stay (six days (IQR: 4-9)) vs (5.5 days (IQR: 4-9)) (P = 0.915).

Secondary MAs (Tier 2): Wang et al. reported a statistically significant reduction in hospital stay with LR (MD = -0.74, 95%CI: -1.20 to -0.28, P = 0.001). However, Zhou et al. (SMD: -0.18, 95%CI: -0.44 to 0.07, I² = 0.0) and Mosquera et al. (SMD: -0.89; 95% CI: -2.26 to 0.48; P = 0.23) found no statistically significant difference.

Progression to Moderate-to-Severe or Severe Acute Pancreatitis

Primary RCTs (Tier 1): In Karki et al., severe progression occurred in 23.07% (6/26) of LR patients compared to 42.30% (11/25) of NS patients, though this did not reach statistical significance (P = 0.11). Lee et al. similarly noted a non-significant reduction (adjusted RR = 0.5, 95%CI: 0.2-1.1).

Secondary MAs (Tier 2): Quantitative synthesis across all three MAs demonstrated statistically significant reductions in disease progression for patients resuscitated with LR: Wang et al. (RR = 0.64, 95%CI: 0.45-0.89, P = 0.008), Zhou et al. (OR = 0.49, 95%CI: 0.25-0.97, I² = 0.0), and Mosquera et al. (RR = 0.63, 95% CI: 0.40-0.98, P = 0.04).

ICU Admission Rate

Four studies specifically evaluated ICU admission rates.

Primary RCTs (Tier 1): Lee et al. reported that the rate of ICU admission in the LR group was less than half that of the NS group, achieving statistical significance (adjusted RR = 0.4, 95% CI: 0.2-0.9).

Secondary MAs (Tier 2): Pooled estimates consistently confirmed a statistically significant reduction in ICU admissions with LR: Wang et al. (RR = 0.39, 95%CI: 0.18-0.85, P = 0.02), Zhou et al. (OR = 0.33, 95% CI: 0.13-0.81, I² = 0.0), and Mosquera et al. (RR = 0.39; 95% CI: 0.18-0.85, P = 0.02).

Local Complications

Local pancreatic complications (e.g., pancreatic necrosis, fluid collections, pseudocysts) occurred less frequently in the LR arm across all studies.

Primary RCTs (Tier 1): Lower incidence rates were observed with LR in Choosakul et al. (26.08% (6/23) vs. 37.5% (9/24), P = 0.74), Karki et al. (23.1% (6/26) vs. 28.0% (7/25), P = 0.68), and Lee et al. (adjusted RR = 0.3, 95%CI: 0.1-1.5), though single-trial sample sizes were underpowered to reach individual statistical significance (P > 0.05).

Secondary MAs (Tier 2): Upon meta-analytic pooling, LR demonstrated a statistically significant reduction in local complications in both Wang et al. (RR = 0.63, 95%CI: 0.42-0.94, P = 0.02) and Zhou et al. (OR = 0.42, 95% CI: 0.20-0.88, I² = 0.0).

Discussion

Interpretation of Findings

To interpret the clinical implications of this review without unit-of-analysis confusion or double-counting, findings must be evaluated across two distinct tiers of evidence: Tier 1 primary RCTs and Tier 2 secondary MAs.

At the primary trial level (Tier 1), individual RCTs (e.g., Lee et al., Choosakul et al., Karki et al.) evaluated direct, unpooled patient cohorts. At the 24-hour mark, Choosakul et al. and Karki et al. found LR to be more effective in reducing SIRS score after 24 hours. Lee reported a lower baseline prevalence of SIRS (26%) in patients than expected (50%), lowering the power of the study; hence, no statistical difference in SIRS was reported at 24 hours. These trials demonstrated consistent early physiological benefits in patients resuscitated with LR compared to NS, specifically evidenced by lower C-reactive protein (CRP) levels and accelerated attenuation of SIRS at 24 hours.

At the secondary synthesis level (Tier 2), published MAs (Wang et al., Zhou et al., and Mosquera et al.) consistently demonstrated statistically significant pooled reductions in ICU admission rates, progression to moderate-to-severe pancreatitis, and local complications. However, pooled findings for early systemic inflammatory parameters were nuanced across MAs. 

SIRS prevalence: Only Wang et al. demonstrated a statistically significant reduction in 24-hour SIRS in the LR group (P < 0.05), whereas Zhou et al. and Mosquera et al. observed non-significant differences for 48-hour SIRS persistence (OR = 0.70 and RR = 0.83, respectively). 

Changes in CRP levels: Mosquera et al. demonstrated a statistically significant reduction in 48-hour CRP levels (SMD = -3.91, P < 0.00001), whereas CRP outcomes were unreported in Wang et al. and Zhou et al.

Crucially, because these secondary MAs synthesize substantially overlapping subsets of the same primary RCTs, their reported effect estimates represent pooled validation of shared trial cohorts rather than additive independent patient populations. 

Mortality benefits were ambiguous because of sparse data on mortality.

The physiological rationale supporting the advantages of LR over NS lies in acid-base homeostasis. High-volume resuscitation with NS delivers a supra-physiological chloride, which frequently precipitates in hyperchloremic metabolic acidosis. Conversely, LR provides a balanced electrolyte profile and a lactate buffer that metabolizes into bicarbonate, maintaining neutral pH, mitigating intracellular acinar injury, and dampening systemic inflammation [27]. Also, it is seen in studies in animal models that LR recovers base excess (BE), which is an indicator of shock, more effectively than NS after fluid loss [28].

Comparing Findings of This Review With Existing Literature

Our study included RCTs and MAs conducted within the past 10 years, with a primary focus on SIRS after 24 hours of fluid therapy. Also, to ensure the findings were representative of a general AP population, studies evaluating critically ill patients were excluded.

Our review aligns closely with the published MAs. Similar mixed results were obtained in Costea et al.'s systematic review, with only some studies reporting LR being more effective than NS in reducing SIRS at 24 hours [4]. One of the biggest MAs with a 1,500 sample size did not find a significant difference between the two groups in SIRS at 24 hours, 48 hours, or 72 hours [29]. It also reported benefits of LR similar to those reported in our study in reducing ICU admissions. In addition, Zhao et al. also found favorable evidence for LR in reducing moderate-severe to severe pancreatitis, the incidence of local complications, and a shorter LOH stay.

The largest clinical trial to date conducted on AP, the WATERLAND trial, further clarifies crystalloid choice: while LR did not show a statistically significant reduction in progression to severe pancreatitis compared to NS, it significantly reduced 24-hour and 48-hour SIRS rates, CRP levels, and acute kidney injury [30]. Beyond crystalloid choice alone, our findings must be contextualized alongside resuscitation intensity and recent clinical trial data. While our review focused on fluid selection (LR vs. NS), the landmark WATERFALL trial evaluated the fluid rate, demonstrating that aggressive resuscitation increases the volume overload without clinical benefit compared to moderate resuscitation [31]. Most recently, a 2026 component network MA disaggregated resuscitation rate and fluid type across RCTs, concluding that a non-aggressive resuscitation strategy specifically using LR provides the optimal balance of safety and clinical efficacy [32]. Together, these recent studies reinforce our findings while demonstrating that the therapeutic benefit of LR is maximized when delivered within a controlled, non-aggressive fluid protocol.

Implications

The findings of this review suggest that LR is the more favorable crystalloid for early resuscitation in adults with AP. The most persuasive evidence is reduced ICU admission and improved inflammatory markers earlier on fluid resuscitation. For mixed benefits in other outcomes, larger, multicenter studies with consistent methods are needed to clarify the effect.

Limitations of the Review

This review is limited by the small size of the evidence base, low quality of evidence, overlap of studies included in our finalized MAs, and the modest number of randomized trials.

All three of the included MAs already had two or three of this study’s finalized RCTs. The six included studies together still represent only 431 different patients, because the evidence base is still relatively small, the estimates are vulnerable to imprecision. Also, several outcomes were reported inconsistently or in only a subset of trials. Overall quality of evidence for SIRS at 24 hours, as reported in Zhou and Mosquera's MAs, was very low and low, respectively. This review was also not prospectively registered on PROSPERO.

There was also methodological heterogeneity. The trials differed in blinding, fluid protocol, resuscitation targets, and timing of reassessment, which likely contributed to the mixed SIRS findings. That is why the MA was not performed in this study.

## Conclusions

This systematic review evaluated whether LR offers superior clinical outcomes compared to NS during fluid resuscitation in adults with AP. Overall, LR appears favorable for select early outcomes, such as short-term inflammatory markers (early SIRS at 24 hours and CRP reduction) and reduced ICU admissions in pooled analyses. However, SIRS findings are largely non-significant beyond 24 hours, and a clear clinical benefit in major outcomes, such as overall mortality and LOH stay, remains unproven. These conclusions are limited by sparse mortality data, small sample sizes, and study overlap across the current literature. While LR demonstrates potential early advantages, well-powered, multicenter RCTs with standardized resuscitation protocols are necessary to determine its definitive impact on hard clinical outcomes.

## References

[1] Tenner S, Vege SS, Sheth SG (2024). American College of Gastroenterology guidelines: management of acute pancreatitis. Am J Gastroenterol.

[2] Biberci Keskin E, Taşlıdere B, Kiremitçi S, Köker İH, Toluk Ö, Şentürk H (2025). Long-term all-cause mortality after acute pancreatitis: median 7-year follow-up. Turk J Gastroenterol.

[3] Karakayali FY (2014). Surgical and interventional management of complications caused by acute pancreatitis. World J Gastroenterol.

[4] Costea CN, Pojoga C, Seicean A (2025). Advances in the management of fluid resuscitation in acute pancreatitis: a systematic review. Diagnostics (Basel).

[5] Wang CF, Tariq A, Chandra S (2025). Acute pancreatitis. StatPearls [Internet].

[6] Dawson A, Karunakaran M, Sharma ZD, Ullah S, Barreto SG (2023). Fluid resuscitation in the early management of acute pancreatitis - evidence from a systematic review and meta-analysis. HPB (Oxford).

[7] Crockett SD, Wani S, Gardner TB, Falck-Ytter Y, Barkun AN (2018). American Gastroenterological Association Institute guideline on initial management of acute pancreatitis. Gastroenterology.

[8] Takada T, Isaji S, Mayumi T (2022). JPN clinical practice guidelines 2021 with easy-to-understand explanations for the management of acute pancreatitis. J Hepatobiliary Pancreat Sci.

[9] Goodchild G, Chouhan M, Johnson GJ (2019). Practical guide to the management of acute pancreatitis. Frontline Gastroenterol.

[10] Arvanitakis M, Dumonceau JM, Albert J (2018). Endoscopic management of acute necrotizing pancreatitis: European Society of Gastrointestinal Endoscopy (ESGE) evidence-based multidisciplinary guidelines. Endoscopy.

[11] (2013). IAP/APA evidence-based guidelines for the management of acute pancreatitis. Pancreatology.

[12] Wu BU, Hwang JQ, Gardner TH (2011). Lactated Ringer's solution reduces systemic inflammation compared with saline in patients with acute pancreatitis. Clin Gastroenterol Hepatol.

[13] de-Madaria E, Herrera-Marante I, González-Camacho V (2018). Fluid resuscitation with lactated Ringer's solution vs normal saline in acute pancreatitis: a triple-blind, randomized, controlled trial. United European Gastroenterol J.

[14] Guzmán-Calderón E, Diaz-Arocutipa C, Monge E (2022). Lactate Ringer's versus normal saline in the management of acute pancreatitis: a systematic review and meta-analysis of randomized controlled trials. Dig Dis Sci.

[15] Hong J, Li Q, Wang Y, Xiang L, Zhou Y, Fan M, Lin R (2024). Comparison of fluid resuscitation with lactate Ringer's versus normal saline in acute pancreatitis: an updated meta-analysis. Dig Dis Sci.

[16] Banks PA, Bollen TL, Dervenis C (2013). Classification of acute pancreatitis--2012: revision of the Atlanta classification and definitions by international consensus. Gut.

[17] Ouzzani M, Hammady H, Fedorowicz Z, Elmagarmid A (2016). Rayyan-a web and mobile app for systematic reviews. Syst Rev.

[18] Shea BJ, Reeves BC, Wells G (2017). AMSTAR 2: a critical appraisal tool for systematic reviews that include randomised or non-randomised studies of healthcare interventions, or both. BMJ.

[19] Sterne JAC, Savović J, Page MJ (2019). RoB 2: a revised tool for assessing risk of bias in randomised trials. BMJ.

[20] Wang Z, Shi K, Mo S, Liu Z, Yao J (2024). A meta-analysis of lactate Ringer's solution versus normal saline in the treatment of acute pancreatitis. Gastroenterol Hepatol.

[21] Zhou S, Buitrago C, Foong A (2021). Comprehensive meta-analysis of randomized controlled trials of lactated Ringer's versus normal saline for acute pancreatitis. Pancreatology.

[22] Mosquera FEC, Benítez EC, Benavides MCC, Muñoz JEC, Castañeda CA, Liscano Y (2025). Fluid resuscitation with lactated Ringer vs. normal saline in acute pancreatitis: a systematic review and meta-analysis of clinical trials. Diseases.

[23] Lee A, Ko C, Buitrago C, Hiramoto B, Hilson L, Buxbaum J (2021). Lactated Ringers vs normal saline resuscitation for mild acute pancreatitis: a randomized trial. Gastroenterology.

[24] Choosakul S, Harinwan K, Chirapongsathorn S, Opuchar K, Sanpajit T, Piyanirun W, Puttapitakpong C (2018). Comparison of normal saline versus lactated Ringer's solution for fluid resuscitation in patients with mild acute pancreatitis, a randomized controlled trial. Pancreatology.

[25] Karki B, Thapa S, Khadka D (2022). Intravenous Ringers lactate versus normal saline for predominantly mild acute pancreatitis in a Nepalese tertiary hospital. PLoS One.

[26] Page MJ, McKenzie JE, Bossuyt PM (2021). Updating guidance for reporting systematic reviews: development of the PRISMA 2020 statement. J Clin Epidemiol.

[27] Rout P, Patel P, Davis D (2025). Crystalloid solutions in intravenous fluid therapy. StatPearls [Internet].

[28] Martini WZ, Cortez DS, Dubick MA (2013). Comparisons of normal saline and lactated Ringer's resuscitation on hemodynamics, metabolic responses, and coagulation in pigs after severe hemorrhagic shock. Scand J Trauma Resusc Emerg Med.

[29] Zhao T, Kang Z, Zhang Q (2025). Lactated Ringer's solution versus saline fluid resuscitation for reducing progression to moderate-to-severe acute pancreatitis: a systematic review and meta-analysis. Int J Surg.

[30] Guilabert L, Cárdenas-Jaén K, Vaillo-Rocamora A (2024). Normal saline versus lactated Ringer's solution for acute pancreatitis resuscitation, an open-label multicenter randomized controlled trial: the WATERLAND trial study protocol. Trials.

[31] de-Madaria E, Buxbaum JL, Maisonneuve P (2022). Aggressive or moderate fluid resuscitation in acute pancreatitis. N Engl J Med.

[32] D'Ambra V, Ricci C, Ingaldi C, Alberici L, Eusebi LH, De Madaria E, Casadei R (2026). Non-aggressive lactated Ringer's resuscitation improves outcomes and has a favorable safety profile in acute pancreatitis: a comprehensive systematic review and component network meta-analysis of randomized controlled trials using GRADE and CINeMA approaches. Pancreatology.

